# Divergent evolution of synchronous gastric ulcers in a high-risk patient: healing vs malignant transformation

**DOI:** 10.1093/gastro/goag077

**Published:** 2026-07-16

**Authors:** Hanning Xu, Haoyang Duan, Guanyi Liu, Yongchen Ma, Jixin Zhang, Han Yan, Chong Liu, Haowa Wu, Xudong Zhao, Weidong Nian, Long Rong

**Affiliations:** Endoscopy Center, Peking University First Hospital, Beijing 100034, P. R. China; College of Medicine, Wuhan University of Science and Technology, Wuhan 430065, Hubei, P. R. China; Endoscopy Center, Peking University First Hospital, Beijing 100034, P. R. China; Department of Chemistry, New York University, New York, NY 10012, United States; Endoscopy Center, Peking University First Hospital, Beijing 100034, P. R. China; Endoscopy Center, Peking University First Hospital, Beijing 100034, P. R. China; Department of Pathology, Peking University First Hospital, Beijing 100034, P. R. China; Endoscopy Center, Peking University First Hospital, Beijing 100034, P. R. China; Endoscopy Center, Peking University First Hospital, Beijing 100034, P. R. China; Endoscopy Center, Peking University First Hospital, Beijing 100034, P. R. China; Endoscopy Center, Peking University First Hospital, Beijing 100034, P. R. China; Endoscopy Center, Peking University First Hospital, Beijing 100034, P. R. China; Endoscopy Center, Peking University First Hospital, Beijing 100034, P. R. China

## Introduction

Early gastric cancer (EGC) is often detected within or adjacent to chronic ulcers with constant injury and repair. Therefore, vigilant endoscopic surveillance with biopsies is crucial for early detection [1]. Endoscopic submucosal dissection (ESD) is the primary curative treatment for EGC, but management strategies depend on the invasion depth. Invasion >500 µm is a well-established benchmark for substantial risk of occult metastases [2]. We present a case highlighting this dilemma: a 70-year-old male patient whose gastric ulcers progressed to EGC with 1,000 µm invasion depth, presenting complex management decisions.

## Case report

The 70-year-old male patient had a 50-year history of tobacco and alcohol use (ceased 2 years prior). He had been diagnosed with stage T2N2M0 right upper lung squamous cell carcinoma, for which radiotherapy had been completed. He also had a 2-year history of antineutrophil cytoplasmic antibody-associated systemic vasculitis with renal failure, and he was managed on hemodialysis. The patient’s MPO serology was negative in November 2023 and February 2024, but it rose from 23 RU/mL to 148 RU/mL by July 2025. He had hypertension for over 30 years, and he did not receive antihypertensives during the 2 years of hemodialysis. He had type 2 diabetes with diabetic retinopathy for > 15 years, which was treated with insulin. He had hyperlipidemia and carotid stenosis for over 15 years, for which he took atorvastatin. He also had coronary artery disease and unstable angina for > 10 years, and he was on aspirin 100 mg daily, which was withheld for the perioperative period (7 days) prior to both ESD procedures.

Regarding his gastric disease-related history, he had a concurrent *Helicobacter pylori* infection; however, eradication therapy was withheld due to multiple comorbidities, including stage 5 chronic kidney disease. Proton pump inhibitor therapy was administered continuously for 8 weeks following each ESD procedure and was used intermittently during episodes of upper abdominal discomfort or acid reflux thereafter. There was no family history of gastrointestinal malignancies.

The patient’s digestive clinical course started with an emergency endoscopy on 12 December 2023 because of melena. Two ulcers were detected: a 3 × 4 cm lesion extending from the angle to the antrum-body junction and a second 4 × 5 cm lesion near the cardia.

During surveillance endoscopy in March 2024, a red scar was observed near the cardia. A portion of the pre-existing angular scar at the gastric angle presented as a white scar, while another portion located at the antrum-body junction showed a red ulcer scar. Histopathological examination of the antrum-body junction showed that partial glands were involved by focal high-grade intraepithelial neoplasia with foci of intramucosal carcinoma, as well as background moderate chronic gastritis with atrophy.

Subsequently, in May 2024, the gastric angle ulcer reexamination showed mild chronic inflammation (activity grade I). At the antrum-body junction, magnifying endoscopy with narrow-band imaging (NBI-ME) identified irregular micro-surface and microvascular structures within the elevated lesion. ESD was performed. The pathology confirmed a moderately differentiated adenocarcinoma (Lauren intestinal type). The majority of the tumor was confined to the lamina propria with focal submucosal invasion reaching 1,000 µm and no lymphovascular and perineural invasion. The background mucosa displayed mild chronic inflammation, moderate intestinal metaplasia, and atrophy. However, the risk of lymph node metastasis (LNM) was considered present. Considering that the EGC exceeded the criteria for invasion depth, surgical intervention was planned. Due to advanced age and comorbidities, the multidisciplinary team deemed surgery as the next step inappropriate.

Surveillance endoscopy in August 2024 revealed a red ulcer scar near the cardia. Biopsy demonstrated only moderate chronic gastritis (activity grade I) with no evidence of neoplasia. A new neoplastic lesion adjacent to the ESD scar was detected in March 2025 and successfully treated with repeat ESD in August 2025. Histopathology confirmed a moderately to well-differentiated intramucosal adenocarcinoma arising in a mucosal background of moderate intestinal metaplasia.

**Figure 1:**
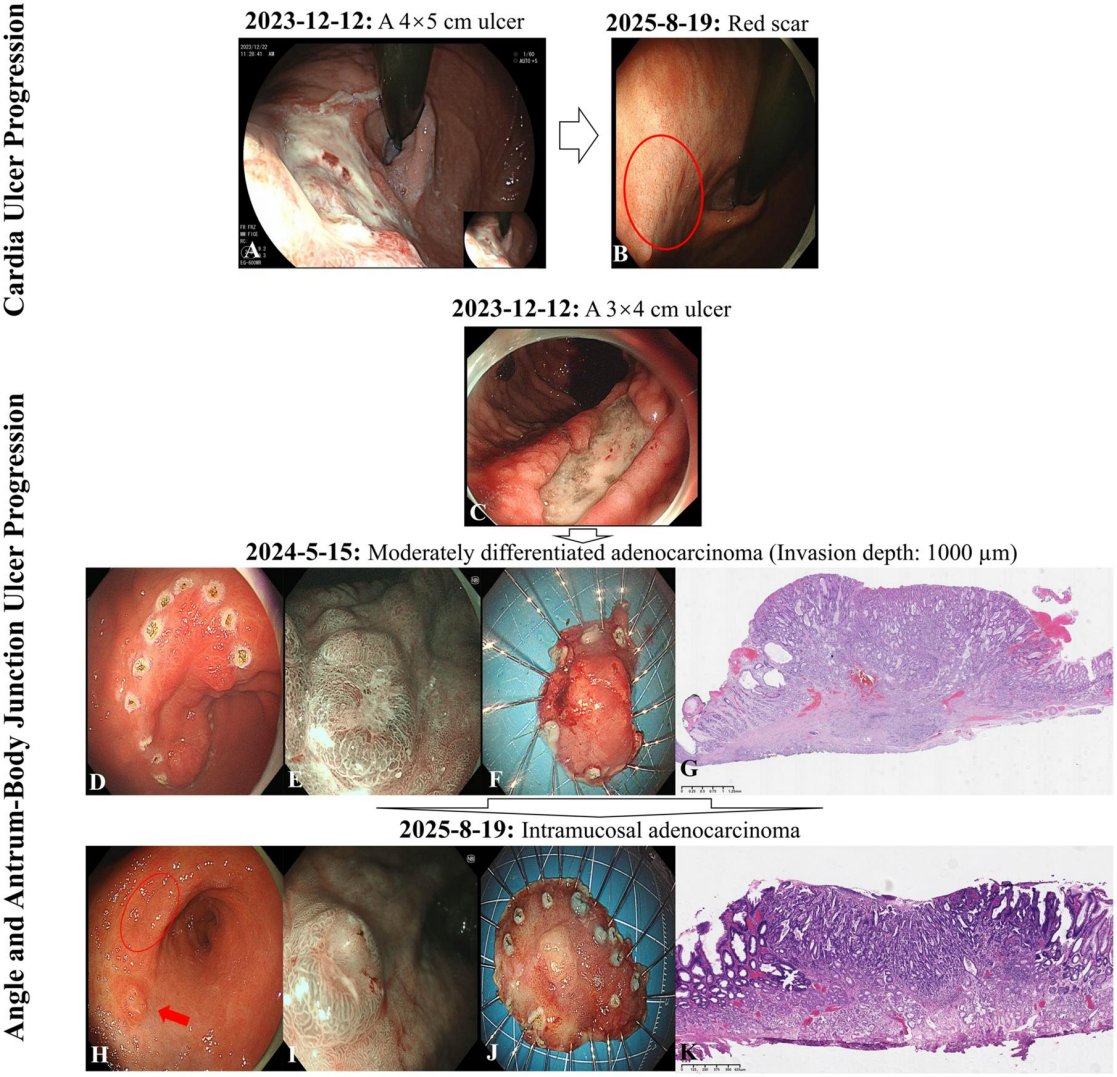
The progression of two large gastric ulcers. (A and B) Progression of a gastric cardia ulcer. (A) WLE on 12 December 2023 showed a 4 × 5 cm ulcer. (B) WLE on August 19, 2025, showed a red scar of ulceration. (C–K) Progression of an ulcer at the gastric angle and antrum-body junction. (C) WLE on 12 December 2023 showed a 3 × 4 cm ulcer. (D–G) NBI-ME and WLE on 15 May 2024, showed an early gastric cancer at part of the red ulcer scar at the antrum-body junction; the area showed an irregular micro-surface structure and microvascular architecture, and histopathological examination showed focal tumor infiltration into the submucosa and lymphovascular invasion. (H–K) NBI-ME and WLE on 19 August 2025, showed a 2.0 × 2.0 cm IIa EGC lesion (arrow) adjacent to the post-endoscopic submucosal dissection scar (circle) at the antrum-body junction with irregular micro-surface structure and microvascular architecture; histopathological examination observed intramucosal carcinoma with a back-to-back or cribriform growth pattern and moderate cellular atypia. WLE, white light endoscopy; NBI-ME, magnifying endoscopy with narrow-band imaging.

During hospitalization for severe pneumonia in December 2025, a follow-up endoscopy performed for the placement of a jejunal feeding tube revealed a well-healed scar at the resection site, with no evidence of residual or recurrent disease. CT showed no gastric wall thickening or signs of locoregional LNM. The chronological progression of the two giant ulcers is demonstrated in Figure 1.

## Discussion and conclusions

The divergent outcomes of the two large synchronous ulcers and the spatial heterogeneity within the single large angulus ulcer may be explained by the concept of “field cancerization” in gastric carcinogenesis [3]. The antrum-body ulcer, anatomically predisposed to *Helicobacter pylori* colonization and further compounded by pulse steroid therapy and long-term aspirin use, likely experienced more intense injury-repair cycles [4]. This created a permissive milieu, which promoted malignant progression [5]. Importantly, the coexistence of healing and malignancy within the single ulcer underscores that comprehensive surveillance of the entire scar field is essential.

Notably, the new neoplastic lesion at the antrum-body junction may support the “field cancerization” effect. Given the relatively longer interval (∼1 year), its location immediately adjacent to the prior scar, and the initially negative resection margins, this lesion is more consistent with a metachronous carcinoma rather than a local recurrence or residual tumor [6]. This underscores the necessity of longitudinal surveillance within these susceptible, high-risk mucosal fields.

Management required balancing discrete risks. ESD for the lesion discovered in March 2024 was initially considered a curative treatment. However, the post-ESD pathology (May 2024) revealed deep submucosal invasion, which upgraded the endoscopic curability to C-2 with a high LNM risk [7]. Importantly, a high statistical risk of LNM is not a certainty for an individual patient. Therefore, for high-surgical-risk patients, a strategy of complete local excision via ESD combined with endoscopic surveillance is a rational approach. The successful treatment of the new neoplastic lesion with repeat ESD in August 2025 underscores the importance of this tailored strategy in select cases [8]. However, the current follow-up duration is relatively short, and longer-term monitoring is necessary to assess the long-term prognosis.

In conclusion, this case offers several insights: (i) ulcers in the setting of chronic gastritis, especially in patients with a convergence of multiple risk factors for gastric cancer, require frequent endoscopic surveillance, (ii) advanced imaging (e.g. NBI-ME) with biopsy is crucial for early and definitive detection [9], and (iii) an individualized risk-benefit assessment is essential; a strategy of ESD combined with endoscopic surveillance is a rational approach for selected patients [10].
